# Evaluating anemia using contrast-enhanced spectral detector CT of the chest in a large cohort of 522 patients

**DOI:** 10.1007/s00330-020-07497-y

**Published:** 2020-11-25

**Authors:** D. Zopfs, M. Rinneburger, D. Pinto dos Santos, R. P. Reimer, K. R. Laukamp, D. Maintz, S. Lennartz, N. Große Hokamp

**Affiliations:** 1 Faculty of Medicine and University Hospital Cologne, Institute for Diagnostic and Interventional Radiology, University Cologne, Kerpener Straße 62, 50937 Cologne, Germany; 2 Department of Radiology, Massachusetts General Hospital, 55 Fruit St, White 270, Boston, MA 02114 USA

**Keywords:** Tomography, X-ray computed, Anemia, Hemoglobin

## Abstract

**Objectives:**

The blood of patients with anemia demonstrates distinctly lower attenuation in unenhanced CT images. However, the frequent usage of intravenous contrast hampers evaluation of anemia. Spectral detector computed tomography (SDCT) allows for reconstruction of virtual non-contrast images (VNC) from contrast-enhanced data (CE). The purpose of this study was to evaluate whether VNC allow for prediction of anemia.

**Methods:**

Five hundred twenty-two patients with CE-SDCT of the chest and accessible serum hemoglobin (HbS) were retrospectively included. Patients were assigned to three groups (severe anemia, moderate/mild anemia, and healthy) based on recent lab tests (≤ 7 days) for HbS following gender and the WHO definition of anemia. CT attenuation was determined using two ROI in the left ventricular lumen and one ROI in the descending thoracic aorta. ROI were placed on CE and copied to VNC. ANOVA, linear regression, and receiver operating characteristics were used for statistic evaluation.

**Results:**

Average HbS was 11.6 ± 2.4 g/dl. Attenuation on VNC showed significant differences between healthy patients, patients with mild/moderate anemia, and severely anemic patients (all *p* ≤ 0.05). Applying cutoffs of 39.2/37.6 HU and 33.6/32.7 HU allowed to differentiate between healthy, mild/moderately, and severely anemic men/women (AUC 0.857/0.833 and 0.879/0.932). A linear relationship between HbS and attenuation on VNC was established (*r*^2^ = 0.54, HbS = − 0.875 + 0.329 × HU).

**Conclusions:**

An approximation of HbS and presence of anemia can be conducted based on simple attenuation measurements in contrast-enhanced SDCT examinations enabled by VNC imaging.

**Key Points:**

*• While the attenuation of blood is a previously described biomarker for anemia in non-contrast images, virtual non-contrast images from spectral detector CT circumvent this limitation and allow for diagnosis of anemia in contrast-enhanced scans.*

*• Attenuation of blood in virtual non-contrast images derived from spectral detector CT shows a moderate correlation to serum hemoglobin levels.*

*• Presence of anemia be estimated in virtual non-contrast images using proposed cutoffs of 39.2 HU and 37.6 HU for men and women, respectively, to differentiate between healthy and anemic patients.*

**Supplementary Information:**

The online version contains supplementary material available at 10.1007/s00330-020-07497-y.

## Introduction

Anemia is defined as a reduced absolute number of circulating red blood cells, represented by a low serum hemoglobin (HbS) concentration or a low hematocrit in clinical practice. Anemia is a highly prevalent disease with a global prevalence of up to 24.8%, whereby women are more frequently affected than men [1, 2]. It is linked to an adverse impact on overall prognosis and negatively affects quality of life, especially in oncologic patient collectives [1, 3]. Thus, it represents a major factor of global disease burden [1, 3, 4]. Routinely, anemia is diagnosed with a laboratory test for HbS using a peripheral blood sample.

Computed tomography (CT) represents an essential diagnostic imaging method in various clinical settings and diseases. Different qualitative findings have been described to diagnose severe anemia in unenhanced CT scans of the thorax, such as the “aortic ring sign” which refers to relatively hypodense blood in comparison to the hyperattenuating wall of the aorta [5–8]. While these findings are dependent on the observer and therefore subject to a higher variability [9], different studies demonstrated distinct correlations between attenuation values in Hounsfield units (HU) of the blood and HbS [10–16]. Yet, all of these studies used unenhanced images to predict anemia respectively HbS. In clinical routine, however, the vast majority of CT examinations are performed with intravenous application of iodinated contrast agent to facilitate a sufficient contrast of the vascular system, lymph nodes, and parenchymatous organs. Following the lack of unenhanced scans in such cases, clinical application of attenuation-based prediction of anemia is often unfeasible.

Dual-energy CT has been extensively researched in the last decade and enables reconstruction of virtual non-contrast (VNC) images, which can be understood as image interpolations from which the iodine-specific signal has been subtracted [17–19]. Such VNC images are available for all clinical implementations of DECT as proprietary reconstruction algorithm based upon three-material decomposition [17, 20]. Previous studies demonstrated a high reliability of dual-energy CT-derived VNC images and excellent correlations between attenuation values in VNC and true non-contrast images irrespective of iodine concentration [20–22]. Hence, our hypothesis was that attenuation measurements in VNC images can be used to detect anemia and predict HbS in contrast-enhanced scans.

## Materials and methods

### Patients

This retrospective single-center study was approved by the institutional review board. Written informed consent was waived as only clinically obtained CT scans and laboratory parameters were used. The picture archiving and communication system and radiological information system were screened by a radiologist for patients with the following inclusion criteria:

1. Patients ≥ 18 years
2. Recent laboratory test with HbS concentration within 7 days prior or after the CT examination
3. Contrast-enhanced, portal venous phase dual-layer spectral detector CT (SDCT) including the chest with standardized examination protocol between January 2017 and September 2019
4. Referral to CT from the department of gastroenterology and hepatology

**Fig. 1:**
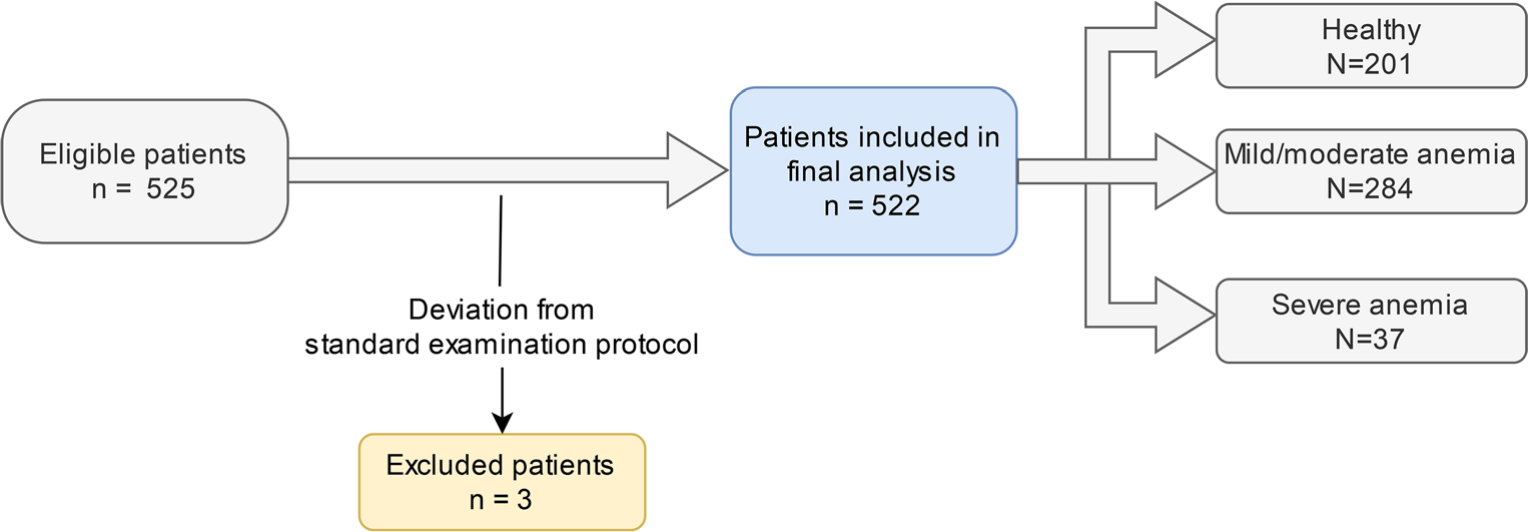
Detailed patient inclusion and exclusion chart

In addition to these portal venous phase scans, a second set of thoracic SDCT in angiographic phase was included in order to evaluate applicability to other contrast injection protocols. A total of 3 scans were excluded due to deviation from the standard scanning protocol. Detailed patient flow is depicted in Fig. 1.

### Image acquisition and reconstruction

All examinations were conducted on a clinical 64-row dual-layer, spectral detector CT (IQon, Philips Healthcare). Patients were scanned in supine position. Following scan presets were used for portal venous scans: tube voltage 120 kVp, enabled tube current modulation (DoseRight 3D-DOM, Philips Healthcare), pitch 0.671, rotation time 0.33 s, collimation 64 × 0.625 mm, matrix 512 × 512. For angiographic scans, the following presets were employed: tube voltage 120 kVp, tube current modulation (DoseRight 3D-DOM, Philips Healthcare), pitch 0.485, rotation time 0.5 s, collimation 64 × 0.625 mm, matrix 512 × 512.

In each patient, 100 ml of iodinated contrast media (Accupaque 350, GE Healthcare) was administered via an antecubital vein using an automated injector (Medrad Stellant CT injection system, Bayer Healthcare). For portal venous phase and angiographic scans, the flow rate was set to 3.0 ml/s and 4.0 ml/s and image acquisition started 50 s and 5 s after reaching a threshold value of 150 HU in the descending aorta, respectively.

Conventional and VNC images were reconstructed in axial plane in a slice thickness of 2 mm and a section increment of 1 mm. Conventional images of portal venous and angiographic scans were reconstructed using a hybrid-iterative reconstruction algorithm (iDose 4, level 3, filter B, Philips Healthcare). VNC images were reconstructed with a dedicated spectral image reconstruction algorithm (Spectral 3, Filter B, Philips Healthcare). As recommended by the AAPM Report No. 204 [23], the size-specific dose estimate (SSDE) was used to report radiation dose. The effective diameter was computed based upon measurements of the anterior-posterior and lateral diameter and used to determine the conversion factor for the 32 cm CTDI_vol_ as per dose report.

### Quantitative image analysis

**Fig. 2:**
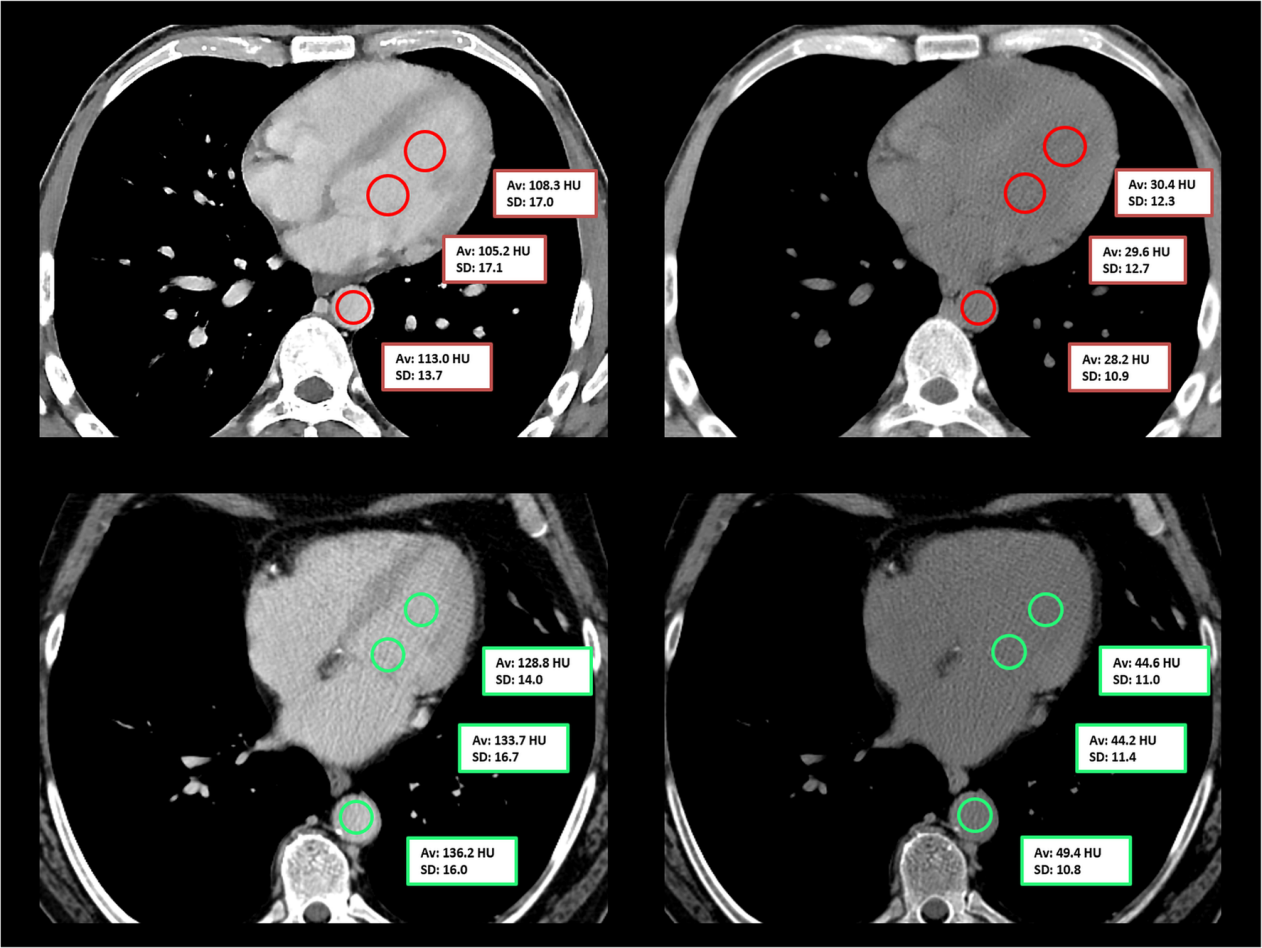
Placement of circular region of interest in the left ventricle and the descending aorta in a 42-year-old anemic male patient (serum hemoglobin 5.6 g/dl; upper row) and in a 59-year-old healthy male patient (serum hemoglobin 14.5 g/dl; lower row) in contrast-enhanced portal venous phase scans (left) and the corresponding virtual non-contrast reconstructions (right). Of note, the “interventricular septum sign” is slightly visible in the virtual non-contrast reconstruction in the upper row

Region of interest (ROI)-based quantitative image analysis was conducted in contrast-enhanced conventional images by a radiologist with 1 year of experience in thoracic imaging. Two ROI were placed in the left ventricle and one ROI was placed in the descending thoracic aorta at the level of the left ventricle (Fig. 2). ROI were drawn as large as possible with a minimum diameter of 1 cm; however, the inclusion of surrounding tissues was avoided. ROI were then copied and pasted onto VNC reconstructions. For each ROI, attenuation and standard deviation (SD) were recorded and all three ROI were averaged for statistical testing.

### Serum hemoglobin

For each patient, HbS levels were retrieved from the hospital information system. Only laboratory tests within ± 7 days of the SDCT exam were included. Three groups, healthy, mild/moderate anemia, and severe anemia, were defined according to the following HbS concentrations as defined by the World Health Organization (WHO) guidelines [24, 25]: Male patients with HbS ≥ 13.0 g/dl and female patients with HbS ≥ 12.0 g/dl were considered healthy. Females with HbS 8.0–11.9 g/dl and males with HbS 8.0–12.9 g/dl were assigned to the mild/moderate anemia group. In all patients, a HbS < 8.0 g/dl was defined as severe anemia.

### Statistic assessment

Statistical analysis was performed using JMP (v14, SAS Institute). Continuous variables are provided as mean ± standard deviation. *T* test, ANOVA, and Wilcoxon test with appropriate adjustments for multiple comparisons (i.e., Tukey-Kramer or Steel-Dwass) have been employed after testing for normal distribution using Shapiro-Wilk test. Furthermore, linear regression was used to evaluate the relationship between hemoglobin and attenuation on CI and VNC images. Receiver operating characteristics (ROC) and Youden’s index were employed to assess performance. Statistical significance was defined as *p* ≤ 0.05.

## Results

### Study population

A total of 522 patients, 301 men and 221 women with a mean age of 63 ± 14 years (range 21–90 years), were included. Of these 522 patients, 485 patients underwent scanning in portal venous phase (chest, abdomen, and pelvis) and 37 patients underwent scanning in angiographic phase (Fig. 1). Mean CTDIvol was 11.06 mGy and average DLP was 823.74 mGy*cm. Average SSDE for the used protocol was 13.2 ± 2.6 mGy, and no significant differences between angiographic and portal venous phase were found (13.2 ± 3.9 mGy and 13.2 ± 2.4 mGy, respectively; *p* = 0.545).

The mean time between the hemoglobin test and the SDCT examination was 1.2 ± 1.5 days (range 0–7 days). HbS levels varied from 5.6–17.6 g/dl, with a mean of 11.6 ± 2.4 g/dl. Mean HbS was slightly higher in men than in women (11.9 ± 2.5 g/dl vs. 11.2 ± 2.1 g/dl). Thirty-seven patients (23 men and 14 women) had severe anemia, 284 patients (162 men and 122 females) had mild to moderate anemia, and 201 patients (116 men and 85 women) were healthy. Detailed patient characteristics are reported in Table 1.

**Table 1:** Detailed patient characteristics. Data are presented as mean ± standard deviation unless otherwise indicated. Patients were assigned to three gender-specific groups according to recent hemoglobin tests and the WHO anemia definition

	Men			Women		
	Healthy (*N* = 116)	Mild/moderate anemia (*N* = 162)	Severe anemia (*N* = 23)	Healthy (*N* = 85)	Mild/moderate anemia (*N* = 122)	Severe anemia (*N* = 14)
Age (years)	60.4 ± 15.9	64.5 ± 11.6	60.3 ± 14.2	65.5 ± 12.8	62.0 ± 15.6	64.6 ± 17.3
Serum hemoglobin (g/dl)	14.4 ± 1.0	10.8 ± 1.5	7.2 ± 0.6	13.3 ± 1.0	10.1 ± 1.1	7.4 ± 0.4
Number of portal venous contrast scans	109	157	22	77	109	11
Number of angiographic contrast scans	7	5	1	8	13	3
Attenuation (contrast enhanced) in HU	151.7 ± 47.4	147.5 ± 29.3	142.2 ± 28.9	177.8 ± 51.2	182.6 ± 43.8	196.7 ± 68.3
Attenuation (virtual non-contrast) in HU	43.2 ± 4.5	37.1 ± 4.2	31.2 ± 4.8	39.9 ± 3.8	34.9 ± 4.9	29.2 ± 2.5

### Differences in portal venous and angiographic phase

Averaged attenuation (HU_Blood_) and corresponding image noise (SD_Blood_) from ROI in the left ventricle and the descending aorta were 156.0 ± 30.8 HU on portal venous images and 227.9 ± 90.3 HU on thoracic angiographies. In corresponding VNC images, mean attenuation was 37.8 ± 5.4 HU and 38.7 ± 6.3 HU, respectively (electronic supplementary material 1). As no significant differences between VNC reconstructions of portal venous and angiographic phase could be established (*p* = 0.780) and HU_Blood_ showed nearly identical correlations to HbS in both angiographic and portal VNC reconstructions (38.7 ± 6.3 HU and 37.8 ± 5.2 HU; *p* = 0.352), VNC attenuation values of portal venous scans and angiographic scans were pooled for further analysis.

### Quantitative CT measurements

**Fig. 3:**
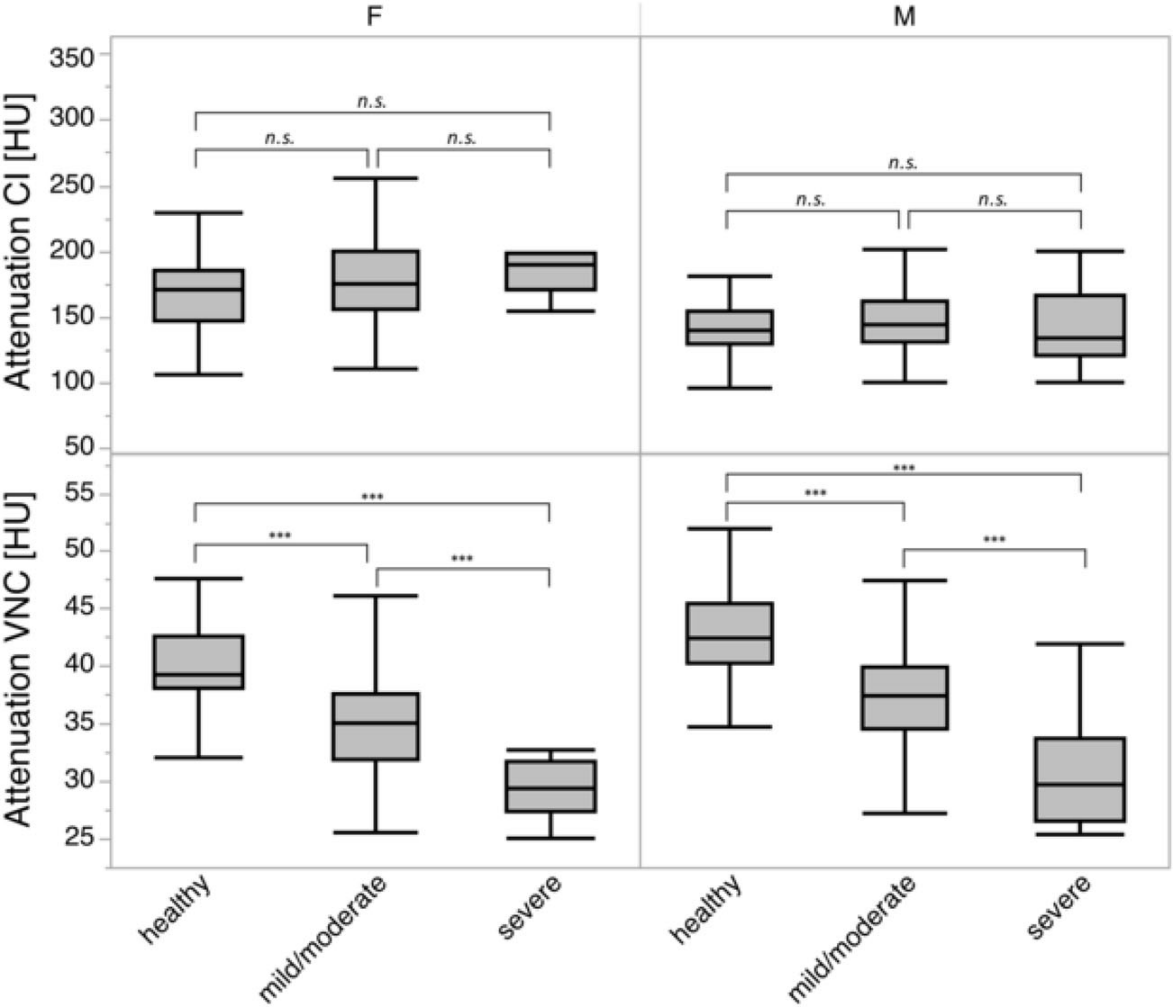
Boxplots of mean attenuation in conventional (CI) and virtual non-contrast images (VNC) in healthy patients and patients with mild/moderate and severe anemia. Note that differences between men and women are likely due to limited sample size in combination with outliers. Significant differences are indicated by asterisks (***p ≤ 0.001, *n.s.*)

Mean HU_Blood_ in contrast-enhanced scans did not differ between groups of patients with severe anemia, mild/moderate anemia, and healthy patients (all *p* > 0.05). However, significant differences regarding HU_Blood_ in VNC images were observed between all groups for both men and women (all *p* ≤ 0.05, Table 1, Fig. 3).

### Differentiation between groups based on VNC measurements

**Fig. 4:**
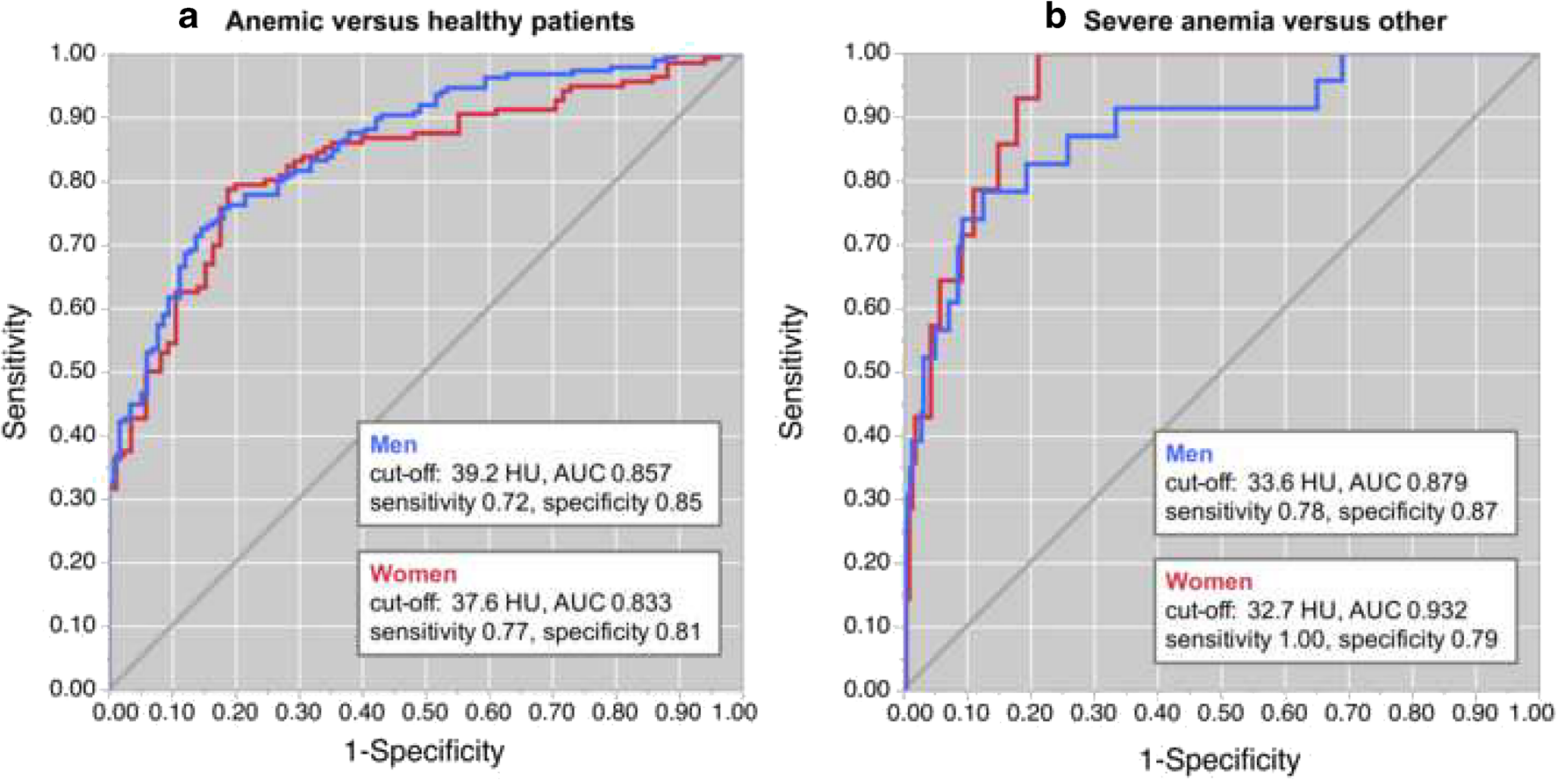
**a** Receiver operator characteristic curves for differentiation between anemic and healthy individuals for men (blue) and women (red). Youden’s index suggests optimal cutoffs of 37.6 HU for women and 39.2 HU for men, respectively. **b** Receiver operator characteristic curves for differentiation between severe anemia and other conditions for men (blue) and women (red). Youden’s index suggests optimal cutoffs of 33.6 HU for women and 32.7 HU for men, respectively

In ROC analysis, differentiation between healthy and any type of anemia yielded an area under the curve (AUC) of 0.857 for men and an AUC of 0.833 for women. Sensitivity and specificity using cutoffs of 39.2/37.6 HU were 0.72/0.77 and 0.85/0.81, respectively (Fig. 4a). To differentiate between severe anemia and other conditions (mild/moderate anemia and/or healthy), an AUC of 0.879 for men and 0.932 for women was obtained. Youden’s index indicated 33.6 HU for men and 32.7 HU for women as optimal thresholds yielding a corresponding sensitivity/specificity of 0.78/0.87 and 1.00/0.79 for men and women, respectively (Fig. 4b). As indicated by quantitative measurements, contrast-enhanced scans performed poor (AUC of 0.544 and 0.490, respectively).

### Correlation between VNC measurements and HbS

**Fig. 5:**
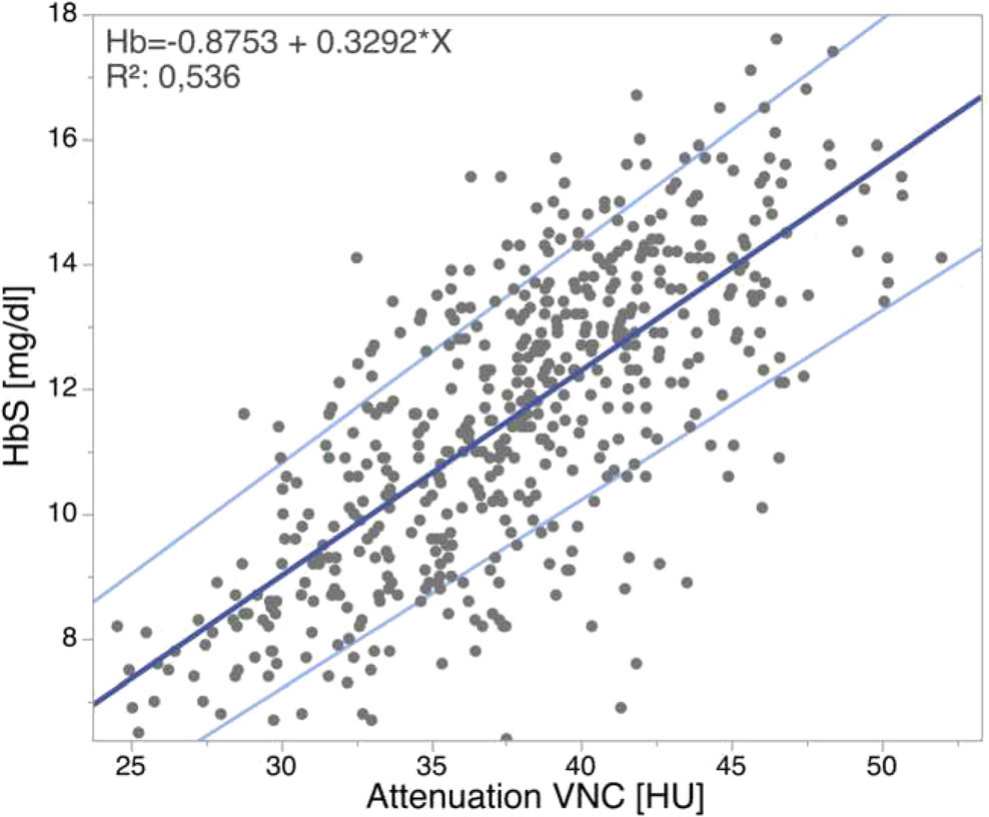
Relation between mean attenuation in Hounsfield units (HU) within the left ventricle and the descending aorta in virtual non-contrast (VNC) images and serum hemoglobin levels in g/dl (HbS). 95% confidence of the estimation equation is shown indicated in light blue

While no significant correlation was found between HU_Blood_ in contrast-enhanced scans and HbS (*p* > 0.05), a linear relationship between HU_Blood_ in VNC images and HbS was observed (*p* ≤ 0.05, Fig. 5). The formula that may be used to estimate the HbS based on ROI measurements in VNC reconstructions is *−* 0.875 + 0.329 × HU (95% confidence interval [*−* 1.886, 0.136] + [0.303, 0.356] × HU).

## Discussion

This study evaluated whether virtual non-contrast (VNC) reconstructions of contrast-enhanced scans obtained from spectral detector CT (SDCT), a dual-layer-based approach to dual-energy CT correlate with serum hemoglobin (HbS) and thus might be helpful for approximating HbS and detect anemia. We found that the attenuation of blood on VNC differs between different degrees of severity of anemia and suggest cutoff values to distinguish between healthy patients and patients with mild/moderate and severe anemia. Furthermore, a formula that may be used to estimate HbS is provided.

Several approaches to dual-energy CT are clinically available. The system used in this study acquires attenuation profiles of low- and high-energy photons on the detector level; therefore, this information is available for all scans without prospective protocol decision [17]. For this scanner, Sauter et al demonstrated that computation of VNC provides accurate estimations of true non-contrast images, irrespective of contrast injection protocol [22]. In an ex vivo study, Duan et al demonstrated the validity of VNC images irrespective of iodine concentration present [26]. Nevertheless, validity of VNC is applicable to soft tissue and parenchymatous organs, only, as several studies indicated limited validity in bone. These reports argue that in bone, calcium might be removed erroneously instead of iodine resulting in false negative values in VNC [18, 22, 27]. The results we found for blood in VNC are well in line with values reported in aforementioned studies [21, 22].

While previous described signs on unenhanced chest CT examinations, such as the “aortic ring sign” and the “interventricular septum sign,” are functional tools in clinical routine and their combination with quantitative attenuation values yielded a very high sensitivity and specificity to discriminate anemic from non-anemic patients in a study from Kamel et al [11], high interobserver variances have been reported for the assessment of such morphologic signs [9]. While the “interventricular septum sign” might be depictable in some anemic patients (Fig. 2), it appears to be a less applicable means of visual analysis in VNC images, likely due to differences in noise and texture [11, 22, 28]. The same accounts for the earlier proposed aortic ring sign [11]. Thus, reliable quantitative measurements validated on large patient collectives are highly desirable to provide confident guidance in the detection of anemia. This idea has been investigated earlier in unenhanced CT scans: Different previous studies described significant correlations between attenuation measurements and HbS [6, 10, 12, 15, 16]. In line with these studies, we found comparable AUC values, sensitivity, and specificity [10, 12]. However, all these aforementioned approaches rely on unenhanced CT images to diagnose anemia, which hampers their practical implementation. In clinical routine, most CT examinations are performed after intravenous contrast media administration, making an assessment of anemia impossible. Jung et al demonstrated the possibility to assess anemia and HbS in a single, unenhanced trigger scan [12]. Although this method may circumvent the issue of contrast-enhanced scans, image noise in trigger scans is high and even aggravated in larger patients, possibly resulting in a greater uncertainty of measurements [12]. Furthermore, the acquisition of unenhanced trigger scans might be started simultaneously with or after contrast agent injection, resulting in uncertainty about true absence of contrast media. As stated by Jung et al, vessel calcifications at the height of the unenhanced trigger scan might alter CT attenuation and consequently represent another limitation. SDCT-derived VNC reconstructions circumvent these obstacles and allow for an anemia diagnosis despite contrast-enhanced images, irrespective of the contrast phase and influences of body weight due to enabled dose modulation.

It needs to be acknowledged that most patients undergoing contrast-enhanced CT will undergo laboratory testing for glomerular filtration rate, and in routine practice, these tests frequently go in hand with a blood count [29–31]. However, not every patient requires a laboratory test and thus analytical data may not be available at the time point of imaging, e.g., in trauma patients [30, 32]. Of note, a general need for a laboratory workup for every patient before intravenous contrast media is not supported by current guidelines, e.g., the manual on contrast media by the American College of Radiology and guidelines by the European Society for Urogenital Radiology [33, 34]. Therefore, non-invasive estimation of HbS may hold a benefit in these patient collectives. Furthermore, a retrospective assessment of HbS may be beneficial in closed prospective trials.

As demonstrated, presence of anemia and/or HbS can be estimated from any thoracic SDCT scan allowing for an opportunistic assessment. Further improvements, e.g., automatic ROI placement, may allow for a fully automated reporting of HbS with every SDCT exam. In our understanding, this may prove particularly beneficial in oncologic patients in which anemia represents an independent predictor of poor prognosis and is further associated with a negative impact on quality of life, longer hospital stays, and thus greater healthcare costs [1, 3].

Apart from the retrospective design, this study has several limitations. First, despite the possibility to assess the anemic state of a patient with only little effort in CT images, the standard tool to assess HbS remains the laboratory test, which a lot of hospitalized patients undergo either way. This is the likely reason why CT-based estimation of HbS using unenhanced scans has not gained clinical relevance so far. Yet, the recent advent of artificial intelligence may provide the opportunity for an increasing opportunistic usage of CT data, such as an automated estimation of HbS with every dual-energy CT scan. Thus, future studies should focus on a fully automated approach to determine anemia in contrast-enhanced SDCT examinations and on adding an estimated HbS to each radiology report. Subsequently, future studies should identify the impact of imaging-based anemia diagnosis on patient care. Second, it needs to be acknowledged that while we included a large cohort of patients undergoing venous phase imaging, the sample size of angiographic phase scans included in this study was smaller; however, as results obtained were unambiguous and in line with earlier reports from literature, we refrained from including further patients and assume transferability to other contrast media application protocols. Third, we did not perform a comparison of VNC with true non-contrast images. Nevertheless, numerous studies demonstrated the accuracy of VNC images in comparison to true non-contrast images [20–22, 35]. Furthermore, formulas and/or cutoff values reported may not be applicable to all patients, e.g., measurements may lead to a false positive result in patients with dyslipidemia. Fourth, all patients were referred to CT from the Department of Gastroenterology and Hepatology; a selection bias cannot be excluded. Last, as a SDCT was used for this study, results are limited to this technological approach; however, as there is an increasing body of evidence demonstrating the accuracy of VNC images for all available dual-energy CT approaches, it can be assumed that similar performance will be observed in these.

To conclude, this study demonstrated that an approximation of serum hemoglobin and anemia can be conducted based on simple attenuation measurements in contrast-enhanced SDCT examinations by means of virtual non-contrast images. Cutoff values to determine a mild, moderate, and severe anemia according to WHO definitions are 39.2 HU and 33.6 HU for men and 37.6 and 32.7 HU for women, respectively.

## Supplementary Information

ESM 1 (DOCX 63 kb)

## Abbreviations

CE: Contrast enhanced
CT: Computed tomography
HbS: Serum hemoglobin
HU: Hounsfield units
SD: Standard deviation
SDCT: Spectral detector CT
VNC: Virtual non-contrast
WHO: World Health Organization

## References

[1] Kassebaum NJ, Jasrasaria R, Naghavi M (2014). A systematic analysis of global anemia burden from 1990 to 2010. Blood.

[2] de Benoist B (2008). Worldwide prevalence of anaemia 1993-2005 of: WHO Global Database of anaemia.

[3] Busti F, Marchi G, Ugolini S, Castagna A, Girelli D (2018). Anemia and iron deficiency in cancer patients: role of iron replacement therapy. Pharmaceuticals (Basel).

[4] McLean E, Cogswell M, Egli I, Wojdyla D, de Benoist B (2009). Worldwide prevalence of anaemia, WHO Vitamin and Mineral Nutrition Information System, 1993-2005. Public Health Nutr.

[5] Wójtowicz J, Rzymski K, Czarnecki R (1983). Severe anaemia: its CT findings in the cardiovascular system. Eur J Radiol.

[6] Lan H, Nishihara S, Nishitani H (2010). Accuracy of computed tomography attenuation measurements for diagnosing anemia. Jpn J Radiol.

[7] Doppman JL, Rienmuller R, Lissner J (1981). The visualized interventricular septum on cardiac computed tomography: a clue to the presence of severe anemia. J Comput Assist Tomogr.

[8] Corcoran HL, Cook DE, Proto AV (1988). Diagnosis of anemia on computed tomography scans of the thorax. J Comput Tomogr.

[9] Title RS, Harper K, Nelson E, Evans T, Tello R (2005). Observer performance in assessing anemia on thoracic CT. AJR Am J Roentgenol.

[10] Zhou Q-Q, Yu Y-S, Chen Y-C (2018). Optimal threshold for the diagnosis of anemia severity on unenhanced thoracic CT: a preliminary study. Eur J Radiol.

[11] Kamel EM, Rizzo E, Duchosal MA (2008). Radiological profile of anemia on unenhanced MDCT of the thorax. Eur Radiol.

[12] Jung C, Groth M, Bley TA (2012). Assessment of anemia during CT pulmonary angiography. Eur J Radiol.

[13] Foster M, Nolan RL, Lam M (2003). Prediction of anemia on unenhanced computed tomography of the thorax. Can Assoc Radiol J.

[14] Chaudhry AA, Gul M, Chaudhry A, Sheikh M, Dunkin J (2015). Quantitative evaluation of noncontrast computed tomography of the head for assessment of anemia. J Comput Assist Tomogr.

[15] Bruni SG, Patafio FM, Dufton JA, Nolan RL, Islam O (2013). The assessment of anemia from attenuation values of cranial venous drainage on unenhanced computed tomography of the head. Can Assoc Radiol J.

[16] Black DF, Rad AE, Gray LA, Campeau NG, Kallmes DF (2011). Cerebral venous sinus density on noncontrast CT correlates with hematocrit. AJNR Am J Neuroradiol.

[17] Hokamp NG, Maintz D, Shapira N, Chang DH, Noël PB (2020). Technical background of a novel detector-based approach to dual-energy computed tomography. Diagn Interv Radiol.

[18] Zopfs D, Lennartz S, Zäeske C et al (2020) Phantom-less assessment of volumetric bone mineral density using virtual non-contrast images from spectral detector computed tomography. Br J Radiol. 10.1259/bjr.20190992 10.1259/bjr.20190992 PMC7217579 32101453

[19] Laukamp KR, Ho V, Obmann VC et al (2019) Virtual non-contrast for evaluation of liver parenchyma and vessels: results from 25 patients using multi-phase spectral-detector CT. Acta Radiol 61(8):1143–1152 10.1177/0284185119893094 31856581

[20] Toepker M, Moritz T, Krauss B (2012). Virtual non-contrast in second-generation, dual-energy computed tomography: reliability of attenuation values. Eur J Radiol.

[21] Ananthakrishnan L, Rajiah P, Ahn R (2017). Spectral detector CT-derived virtual non-contrast images: comparison of attenuation values with unenhanced CT. Abdom Radiol (NY).

[22] Sauter AP, Muenzel D, Dangelmaier J (2018). Dual-layer spectral computed tomography: virtual non-contrast in comparison to true non-contrast images. Eur J Radiol.

[23] AAPM Task Group Size-Specific Dose Estimates (SSDE) in Pediatric and Adult Body CT Examinations: Report No. 204; Available via https://www.aapm.org/pubs/reports/rpt_204.pdf. Accessed 04 Sep 2020

[24] WHO (2011) Haemoglobin concentrations for the diagnosis of anaemia and assessment of severity: Vitamin and Mineral Nutrition Information System. Available via http://www.who.int/vmnis/indicators/haemoglobin.pdf. Accessed 17 Nov 2020

[25] Pasricha S-R, Colman K, Centeno-Tablante E, Garcia-Casal M-N, Peña-Rosas J-P (2018) Revisiting WHO haemoglobin thresholds to define anaemia in clinical medicine and public health. Lancet Haematol 5(2):e60–e62 10.1016/S2352-3026(18)30004-8 29406148

[26] Duan X, Arbique G, Guild J, Xi Y, Anderson J (2018). Technical note: quantitative accuracy evaluation for spectral images from a detector-based spectral CT scanner using an iodine phantom. Med Phys.

[27] Ding Y, Richter A, Stiller W, Kauczor H-U, Weber TF (2019). Association between true non-contrast and virtual non-contrast vertebral bone CT attenuation values determined using dual-layer spectral detector CT. Eur J Radiol.

[28] Große Hokamp N, Gilkeson R, Jordan MK (2019). Virtual monoenergetic images from spectral detector CT as a surrogate for conventional CT images: unaltered attenuation characteristics with reduced image noise. Eur J Radiol.

[29] Davenport MS, Perazella MA, Yee J (2020). Use of intravenous iodinated contrast media in patients with kidney disease: consensus statements from the American College of Radiology and the National Kidney Foundation. Radiology.

[30] Thomsen HS, Morcos SK (2005). In which patients should serum creatinine be measured before iodinated contrast medium administration?. Eur Radiol.

[31] van der Molen AJ, Reimer P, Dekkers IA (2018). Post-contrast acute kidney injury. Part 2: risk stratification, role of hydration and other prophylactic measures, patients taking metformin and chronic dialysis patients. Recommendations for updated ESUR Contrast Medium Safety Committee guidelines. Eur Radiol.

[32] Pantel H, Stensland KD, Hashim J, Rosenblatt M (2017). Is measurement of renal function necessary for all trauma patients before iodinated contrast administration?. Emerg Radiol.

[33] European Society of Urogenital Radiology ESUR Guidelines on Contrast Media; Available via http://www.esur.org/guidelines/en/index.php#a. Accessed 29 Apr 2020

[34] American College of Radiology (2015) ACR manual on contrast media, 10th edn. American College of Radiology, Reston.

[35] Javadi S, Elsherif S, Bhosale P et al (2020) Quantitative attenuation accuracy of virtual non-enhanced imaging compared to that of true non-enhanced imaging on dual-source dual-energy CT. Abdom Radiol (NY) 45(4):1100–1109 10.1007/s00261-020-02415-8 32052130

