# Supplementary material for: Evaluating anemia using contrast-enhanced spectral detector CT of the chest in a large cohort of 522 patients
Source: Eur Radiol. 2020 Nov 25;31(6):4350–7. doi: 10.1007/s00330-020-07497-y (PMC8128794; doi:10.1007/s00330-020-07497-y)

**Electronic Supplementary Material**

**Electronic supplementary material 1**. Left: Boxplots of mean attenuation in virtual non-contrast images (VNC) of angiographic and portal venous scans (left) Differences were not significant (n.s.). Right: Correlation of serum hemoglobin levels (HbS) and attenuation in VNC images of angiographic (r2 = 0.596) and portal venous scans (r2 = 0.536).


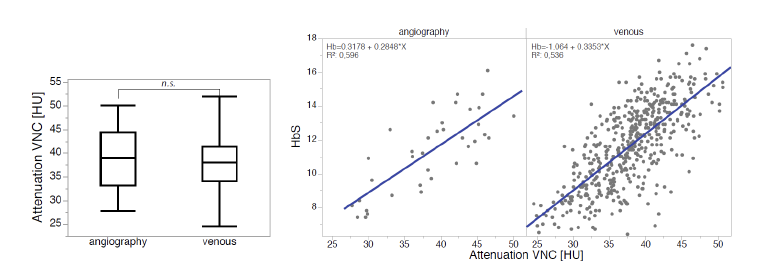

Supplement: Supplementary file 1 — (DOCX 63 kb) [file 330_2020_7497_MOESM1_ESM.docx]
